# Preparation and Adsorption Properties of Lignin/Cellulose Hydrogel

**DOI:** 10.3390/ma16124260

**Published:** 2023-06-08

**Authors:** Xiaoyu Li, Penghui Li, Wei Chen, Jianpeng Ren, Wenjuan Wu

**Affiliations:** 1 College of Light Industry and Food Engineering, Nanjing Forestry University, Nanjing 210037, China; lixiaoyu@njfu.edu.cn (X.L.); cww@njfu.edu.cn (W.C.);; 2 Jiangsu Co-Innovation Center of Efficient Processing and Utilization of Forest Resources, Nanjing Forestry University, Nanjing 210037, China

**Keywords:** porous carbon, adsorption method, dye, phenolic resin, lignin

## Abstract

With the development of global industry, industrial wastewater pollution has caused serious environmental problems, and the demand for green and sustainable adsorbents is increasingly strong in the society. In this article, lignin/cellulose hydrogel materials were prepared using sodium lignosulfonate and cellulose as raw materials and 0.1% acetic acid solution as a solvent. The results showed that the optimal adsorption conditions for Congo red were as follows: an adsorption time of 4 h, a pH value of 6, and an adsorption temperature of 45 °C. The adsorption process was in line with the Langmuir isothermal model and a quasi-second-order kinetic model, which belonged to single molecular layer adsorption, and the maximum adsorption capacity was 294.0 mg/g. The optimal adsorption conditions for Malachite green were as follows: an adsorption time of 4 h, a pH value of 4, and an adsorption temperature of 60 °C. The adsorption process was consistent with the Freundlich isothermal model and a pseudo-second-order kinetic model, which belonged to the chemisorption-dominated multimolecular layer adsorption with the maximum adsorption capacity of 129.8 mg/g.

## 1. Introduction

With the development of global industries, industrial wastewater pollution has caused serious environmental problems, and a part of it belongs to dye wastewater. According to available data, 1–10% of dyes are not used in the dye process, which indicates that this part of dyes is discharged into water by various means [1]. In addition to being toxic, some specific dyes can negatively affect the skin, kidneys, reproductive system, and nervous system and can even cause cancer [2]. Organic dyes are widely used in the dyeing process because of their advantages such as a simple synthetic process, a low cost, and good dyeing performance. In the dyeing process of organic dyes, fabrics need to be washed several times to improve the dyeing fastness, resulting in a large amount of organic dye wastewater, among which Congo red (CR) dye is the representative of anionic dye, and malachite green (MG) is the representative of cationic dye [3]. Dyes in wastewater can generally be removed by chemical, biological, and adsorption methods; biological and chemical methods are effective but costly compared to adsorption methods [4]. Currently, most recognized technologies are using adsorption to remove dyes [5]. However, traditional adsorbent-activated carbon is costly and not suitable for use in industrial production [6]. In contrast, biomass adsorption, due to its biocompatibility and environmental friendliness, is an effective, low-cost method. Lignin, cellulose, and other biomass can be modified into porous materials, and their adsorption principle is the same as activated carbon [7]. Therefore, cellulose and lignin, as typical biomass, have received extensive attention in the study of adsorption methods. As shown in Table 1, it is the adsorption capacity of some common adsorbents for dyes.

Lignin is a complex aromatic compound that generally consists of three benzenepropane substituents, including guaiacyl (G), syringyl (S), and *p*-hydroxyphenyl (H), covalently bound via ether linkages and C-C bonds [8]. Cellulose is a very homogeneous homopolymer consisting of dehydrated glucopyranose units linked together by β-1→4 glycosidic bonds and intramolecular and intermolecular hydrogen bonds linked together in parallel stacks in chains that can form short-range nanocrystalline regions [9,10]. Lignin and cellulose are rich in functional groups such as methyl, methylene, carbonyl, and carboxyl groups [11], and most of them basically have a chelating ability, which facilitates the adsorption of metal ions in water, so they can be used as potential active centers for dye and metal ion adsorption. Among the derivatives of lignin, the most widely used are lignosulfonate, which is present in large quantities in black liquor during the production of paper, and lignosulfonate, which has the merits of low cost, low toxicity, and environmental compatibility. These derivatives have structures that include functional groups such as hydroxyl, carbonyl, and carboxyl groups, and this has also received the attention of a large number of researchers [12].

Hydrogel is a network structure composed of macromolecules with a high water absorption capacity, and its flexibility, elasticity, and permeability are attributed to this high water absorption capacity. Hydrogels are usually formed by chemical crosslinking or physical crosslinking [13]. Owing to their high water retention and their low cost, hydrogels have gained wide interest for their use in sulfonation. However, most hydrogel preparation methods use petroleum-based products as raw materials, which can have a negative impact on the environment. In addition, the preparation process of conventional hydrogels is usually complex, leading to high prices. Driven by the idea of sustainable development, the use of waste biomass, such as cellulose, lignin, and their derivatives, as low-cost materials for the preparation of high-performance hydrogel adsorbents has gained widespread attention [14]. In recent years, relevant applications have been seen in many fields, such as drug transport systems, tissue engineering scaffolds, biosensing, adsorbents, etc. [15]. Lignin composite hydrogels are characterized by obvious porosity, a large specific surface area, high mechanical strength, and chemical stability. Therefore, lignin composite hydrogels can adsorb dye molecules via chemical interaction, electrostatic interaction, and hydrogen bonding [3,16].

Lignin composite hydrogels can be prepared either by physically incorporating lignin into various hydrogels or by chemical crosslinking methods. A relatively simple chemical crosslinking method is the synthesis of hydrogels from lignin by free radical polymerization [17,18]. For example, Ajoy et al. [19] prepared polyacrylic acid hydrogels using lignin sulfonates and Al^3+^, in which the phenolic hydroxyl and catechol groups contained in lignin are susceptible to oxidation to form quinones, and this quinone and Al^3+^/Al^2+^ can induce more free radicals during the redox process, leading to a very rapid polymerization reaction. Juan et al. [20] prepared lignin hydrogels by atom transfer radical polymerization, and these hydrogels showed high removal rates for methylene blue. Li et al. [21] chose carboxymethyl cellulose crystals as raw materials and cysteine as a crosslinking agent to prepare disulfide cross-linked hydrogels, which have a promising future as environmentally friendly adsorbents in solving the problems caused by organic dyes. Based on this, a novel lignin/cellulose hydrogel was prepared by free radical polymerization using lignin, cellulose, and lignosulfonate as the basic raw materials. The hydrogel was freeze-dried to facilitate the adsorption of dyes. 

**Table 1:** Maximum adsorption capacity of different adsorbents for dyes.

Adsorbent	Preparation Method	Dye	Adsorption Capacity (mg/g)	Reference
Biomass lignin-based PVA super-absorbent hydrogel	PVA as template and epichlorohydrin as crosslinking agent.	Methylene blue	179	[22]
Cellulose nanofibrils/alkali lignin/montmorillonite/polyvinyl alcohol Network hydrogel	Adding nano-cellulose, alkali lignin, and montmorillonite into the system of polypropylene-alcohol–water base.	Methylene blue	67.2	[23]
Lignosulfonate ionic hydrogel	Crosslinking with poly(ethylene glycol) diglycidyl ether,	Methylene blue	211	[24]
Cellulose-based hydrogel	the cellulose-based hydrogel was prepared based on the copolymerization of acrylic acid on cellulose materials, with the addition of N,N’-methylene bis-acrylamide as crosslinking agent, and assisted by ammonium persulfate as initiator.	Methylene blue	41.67	[25]
Lignin-based hydroxyethyl cellulose super-absorbent hydrogel	With long-chain hydroxyethyl cellulose as the skeleton, short-chain polypropylene alcohol as the branch chain, lignin as the extension crosslinking agent, and propylene oxide as the crosslinking agent.	Crystal violet	184	[26]
Nano-ZnO-coated cellulose/starch/activated carbon hydrogel	Using beet pulp cellulose/starch/activated carbon as raw material, it was synthesized by crosslinking and ultrasonication.	Methyl orange	72.63	[27]
Carboxymethyl cellulose/chitosan hydrogel	It is prepared by crosslinking carboxymethyl cellulose and chitosan with epichlorohydrin.	Acid orange	100	[28]
Sugar beet pulp cellulose/sodium alginate/iron hydroxide composite hydrogel	Add cellulose, sodium alginate, and iron hydroxide in NaOH/H_2_O as solvent and use epichlorohydrin as crosslinking agent.	Methylene blue	105.93	[29]
carboxymethyl cellulose-g- polyacrylamide/montmorillonite nanocomposite hydrogel	Carboxymethyl cellulose-based graft poly(acrylamide) hydrogel and its nanocomposite with montmorillonite were produced by the free radical method.	Malachite green	158.1	[30]
Cellulose nanocrystal–alginate hydrogel	Mix cellulose nanocrystals with sodium alginate and then squeeze them into cacl_2_ solution.	Methylene blue	256.41	[31]
Double-network gelatin/chitosan hydrogel	Dissolve chitosan in acetic acid aqueous solution, mix it with 20% gelatin aqueous solution, and then add 0.5% glutaraldehyde.	Congo red	221.2	[32]

## 2. Materials and Methods

### 2.1. Source of Materials

Ammonium persulfate (AR), carboxymethyl cellulose (AR), acrylic acid (AR), and N’n-methylene dipropylamine (AR) were supplied by Shanghai Maclin Biochemical Technology Co., Ltd. (Shanghai, China). Lignosulfonate (AR) was supplied by Tixiae (Shanghai) Chemical Industrial Development Co., Ltd. (Shanghai, China).

### 2.2. Fabrication of Lignin/Cellulose Hydrogel

Dissolve 0.25 g of sodium lignosulfonate in 30 mL of deionized water and dissolve 0.75 g and 1 g of cellulose in 10 mL of 0.1% mass fraction of acetic acid solution, separately. The sodium lignosulfonate solution and 0.25 g cellulose acetate solution were slowly fused, and when the solution dissolution was completed, the two solutions were transferred into a 100 mL three-neck flask and stirred well at 30 °C. Then, 10 mL of acrylic acid, 0.1 g of N’N-methylenedipropylamine, and 0.1 g of sodium persulfate were added and reacted under the protection of nitrogen at 60 °C for 3 h. This solution was freeze-dried at −80 °C for 48 h (to test its adsorption properties) and then removed, cut, dried, and ground to finally obtain lignin/cellulose aerogel (LCA) and cellulose aerogel (CA).

See Supplementary Materials for the experimental part of characterization.

### 2.3. Effect of Adsorption Conditions on Adsorption Performance

Adsorption of cellulose hydrogel and lignin/cellulose hydrogel

To investigate the adsorption performance of two adsorbents, cellulose hydrogel and lignin/cellulose hydrogel, a single variable method was adopted for different pH values and adsorbent dosages.

The adsorption performance under different pH conditions was adjusted by 0.1 mol/L HCl and 0.1 mol/L NaOH in the experiment. A total of 20 mg of each adsorbent was added to CR and MG solutions with concentrations of 100 mg/L and pH values of 3, 4, 6, 8, 10, and 12, and the final solutions were placed in conical flasks, and the adsorption temperature was 25 °C. After adsorption, the supernatant was filtered with a 0.22 μm filter membrane, and the supernatant was tested for absorbance by UV spectrophotometer, and if the absorbance was too high, the absorbance was diluted and measured again.

The effect of adsorbent dosage. The dried cellulose hydrogels and lignin/cellulose hydrogels (10 mg, 20 mg, 30 mg, 40 mg, and 60 mg) were accurately weighed to constant weight and placed in 25 mL of CR and MG dye solutions at a concentration of 100 mg/L. The adsorption was carried out at 25 °C for 3 h and 4 h in a thermostatic shaker at 150 rpm. The absorbance after adsorption was measured, and the corresponding adsorption concentration was calculated from the standard curve, and the amount of dye adsorbed and the removal rate were calculated according to the Equations (1) and (2):

(1) $$Q_{t}=(C_{0}-C_{t})V/m$$

(2) $$C\%=\frac{{(C}_{0}-C_{t})}{C_{0}}\times 100$$

where:

Q_t,_ C—dye adsorption capacity, mg/g; dye removal rate, C%;
C_0,_ C_t_—initial concentration of organic dyes; dye concentration after adsorption, mg/L;
V, m—volume of dye to be adsorbed, L; mass of adsorbent, g.

## 3. Results

### 3.1. Morphology and Pore Structure

The morphology and microstructure of the samples were characterized by scanning electron microscopy (SEM).

**Figure 1:**
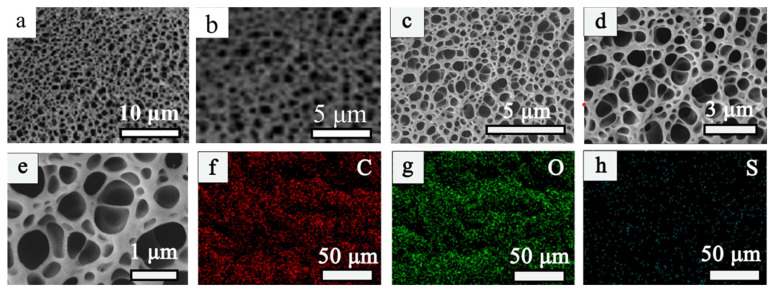
(**a**,**b**) are the electron microscopic scanning of cellulose aerogel; (**c**–**e**) are the electron microscopic scanning of lignin/cellulose aerogel material; (**f**–**h**) are the surface element analysis of C, O, and S on the surface of lignin/cellulose aerogel.

As shown in the diagram, Figure 1a–d show the irregularly shaped nanoscale pores at different magnifications. Comparing the two sets of images, the results show that the pore shape is denser when lignin is added to the cellulose. This is because the addition of lignin will fill the larger pores, which helps to reduce the pore size and special surface area [33]. Therefore, compared to the CA, the LCA shows flexible perforated bones with a uniform passage structure, which is more conducive to ionic diffusion and liquid penetration during dye adsorption. The element mapping technology was used to further study the element distribution of the LCA structure. As shown in the diagram, the findings showed that, in addition to C and O occupying the surface of the material as the main body, a certain amount of S was evenly distributed on the surface of the LCA, which further indicated that lignosulfonate was successfully added to the CA, and the addition of S could improve the adsorption efficiency of dyes to some extent.

### 3.2. Infrared Spectrogram Analysis

**Figure 2:**
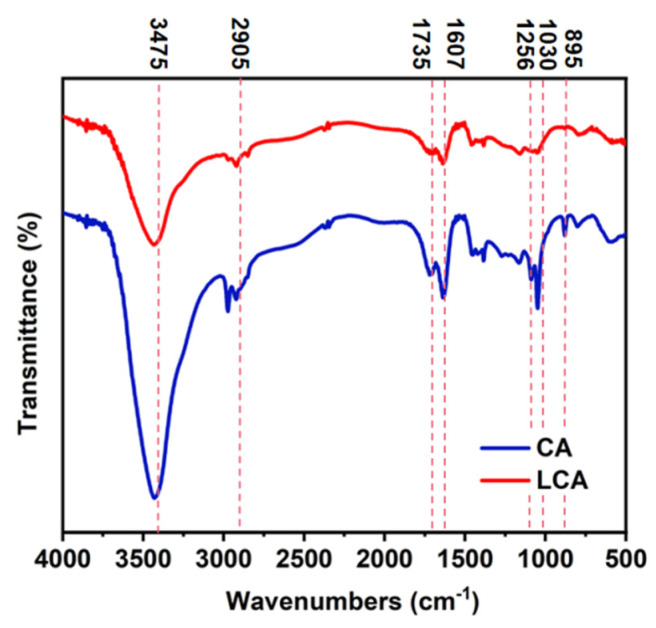
FTIR spectra of LCA and CA.

From Figure 2, it can be seen that cellulose hydrogels and lignin/cellulose hydrogels have -OH stretching vibration peaks and -CH stretching peaks at 3475 cm^−1^ and 2905 cm^−1^, respectively. With the addition of lignin, the absorption strength of the -OH stretching vibration of the LCA at 3475 cm^−1^ weakens. In the processes of lignin adhesion and filling in the three-dimensional network structure of the cellulose, the hydrogen bond between cellulose chains was broken [3,34], and the peak value at 1607 cm^−1^ indicated that both hydrogels contained water-adsorbed -OH [35]. The peak of 1735 cm^−1^ indicates the existence of a vibration peak of -C=O. As shown in Figure 2, the absorption intensity of the LCA is significantly weakened here, which also proves that the lignin is embedded into the cellulose. It is worth noting that the absorption peak of 1256 cm^−1^ is attributed to the aromatic skeleton vibration of lignin, and the absorption peak of the CA at 1256 cm^−1^ is not observed in the FTIR spectrum of the LCA, indicating that the LCA does have more lignin than the CA. The vibration around 1030 cm^−1^ corresponds to the -C-C-, -C-OH, and C-H rings of cellulose, and the absorption peak of the β-D glucoside bond in the cellulose at 895 cm^−1^ is the characteristic structural peak of cellulose type Ⅱ [36].

### 3.3. Thermogravimetric Analysis

**Figure 3:**
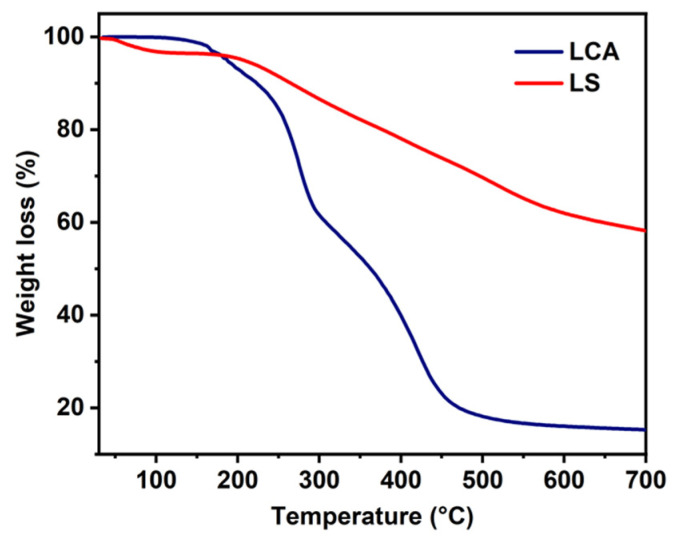
TG curves of LCA and LS.

As shown in Figure 3, the two samples of the lignin sulfonate and the lignin/cellulose hydrogel show different thermal cleavage properties. LS begins to depolymerize and decompose the small molecules on its surface after 150 °C, and the most of the lost weight occurs at 261 °C. The lost weight of LS at 200–400 °C is caused by the separation of dopants and the departure of oligomers or byproducts (*p*-diphenols and quinones) [37,38]. The pyrolysis of the LCA is mainly separated into three steps. The first stage is from the initial temperature to 160 °C. At this stage, the small weight loss of the sample is caused by the evaporation of the water contained in the material. The second stage mainly occurs between 160 °C and 450 °C. This the rapid weight loss stage, which is the main thermal decomposition stage of the sample’s decomposition into small molecules and gaseous products. The third stage occurs above 450 °C. This stage is mainly when the residue is further degraded to gas and residual char [39]. In comparison, the crosslinking polymerization of lignin with cellulose did not improve the thermostability of lignin sulfonate but accelerated the thermal degradation of lignin as the temperature increased.

### 3.4. Adsorption–Desorption Analysis

**Figure 4:**
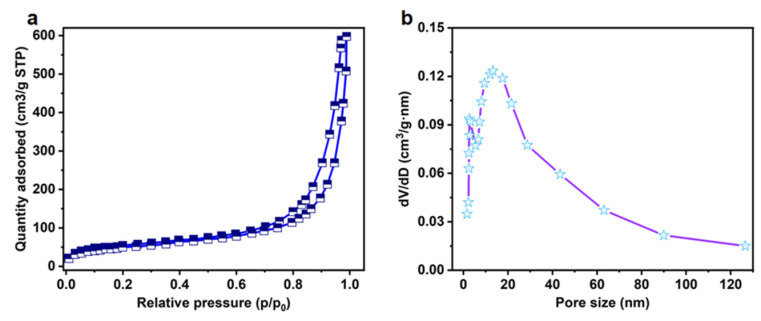
N_2_ adsorption–desorption curve of LCA (**a**); mesoporous and micropore pore size distribution of LCA (**b**).

Figure 4 shows the adsorption and desorption curves and pore size distribution of the lignin/cellulose hydrogels. The specific surface area and pore volume of the sample are 394.3 m^2^/g and 0.785 cm^3^/g, respectively. As shown in the figure, the nitrogen adsorption capacity increases slowly in the low voltage portion of the adsorption profile (0–0.1), increases linearly in the intermediate pressure section (0.2–0.8), and increases rapidly in the high-pressure part (0.8–1.0). This is consistent with the properties of the Langermuir type II adsorption isotherm [17]. From Figure 4a, it can be seen that the hysteresis loop appears at the larger position of P/P_0_, and, combined with Figure 4b, it can be presumed that the material has a more mesoporous structure, with macropores, capillary condensation, and multilayer adsorption on the surface of the material.

### 3.5. XRD Analysis

**Figure 5:**
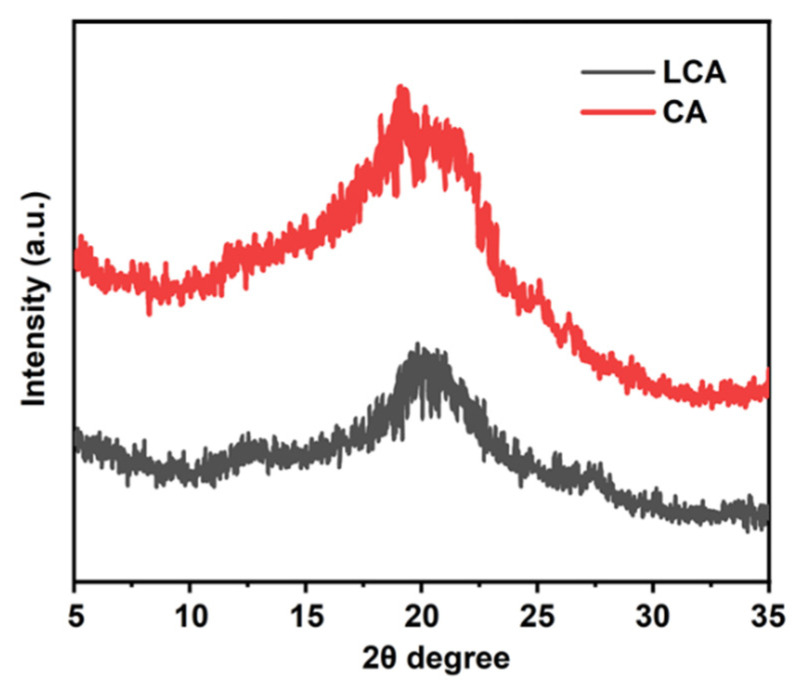
XRD diffraction patterns of LCA and CA.

Figure 5 shows the XRD patterns of the LCA and the CA. As you can see from the chart, the XRD patterns of both remain basically the same. The peaks at 22° in pure cellulose disappeared, and a new peak appeared at 2θ = 20°, indicating that the cellulose crystal structure changed from parallel cellulose chains (cellulose type I) to an anti-parallel arrangement (cellulose type II). This indicated that the crystal structure of the cellulose would be affected in the whole preparation process of the hydrogel [40]. Compared with the CA, after adding lignin, the diffraction peak of the LCA decreases with increases in lignin [23]. By comparing the CA with the LCA, it can be inferred that the introduction of lignin and the crosslinking polymerization reaction affected the crystal structure of the original cellulose [41]. These results were consistent with the FTIR results, which indicated that lignin was embedded into the cellulose to form composite hydrogels during the preparation of the LCA hydrogels.

### 3.6. XPS Analysis

**Figure 6:**
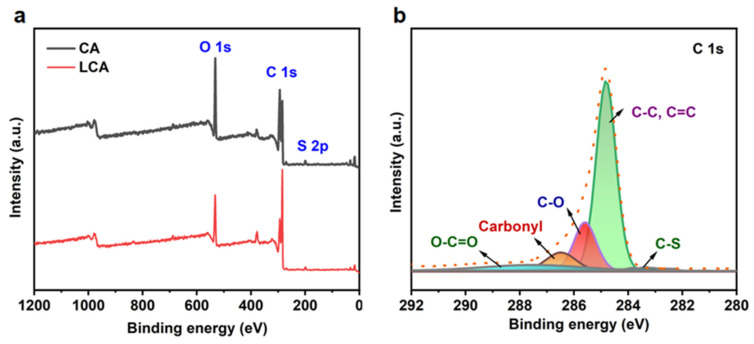
XPS full spectrum (**a**) and C 1s fine spectrum (**b**) of LCA and CA.

To investigate the chemical composition of the samples and to determine the chemical states of the O, C, and S, the XPS full spectra of the LCA and CA were measured by X-ray photoelectron spectroscopy. As shown in Figure 6a, the photoelectron peak of O 1s is at 532.9 eV, which may be attributed to C-O-H or C-O-C; the photoelectron peak of C 1s is at 284.6 eV; and the photoelectron peak of S 2p is at 168.1 eV [3]. This indicates that both the LCA and CA contain O, C, and S, with a lower content of S. Figure 6b depicts the regional XPS spectrum of the C 1s of the sample. The region of C 1s was fitted to the different components of the O-C=O, C-O, C-C, C=C, and C-S bonds corresponding to the sample with photoelectron peaks at 288.5 eV, 285.6 eV, 284.6 eV, and 283.5 eV, respectively. Among them, the O-C=O and C-S bonds were found in the added lignin samples, and S was present in the lignin sulfonic acid group, indicating that lignin was successfully crosslinked with cellulose [14].

## 4. Adsorption Studies of LCA and CA on Congo Red and Malachite Green

### 4.1. The Influence of pH on Adsorption Performance

The adsorption of dye is a complex process, and the pH of the solution is one of the important parameters affecting the adsorption of CR and MG dyes, which affects the concentration of ions on the functional groups of the adsorbent and the ionization of the adsorbent during the reaction [42]. To investigate the influence of pH on the adsorption of CR and MG by LCA materials, the initial pH range of 3–12 was used as a variable condition for the experiments [3,14].

**Figure 7:**
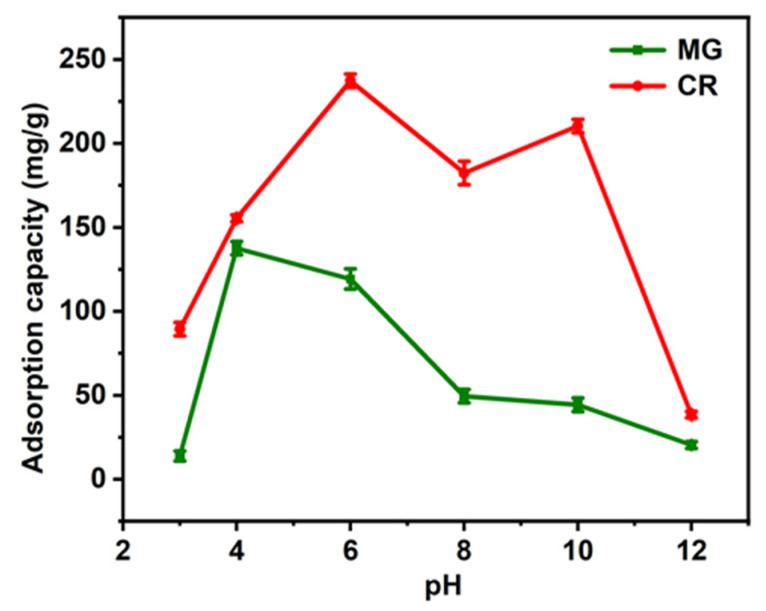
Effect of pH on the amount of adsorption of MG and CR.

As shown in Figure 7, the adsorption of MG by the LCA increased with pH < 4 and decreased with pH > 4. The decrease in adsorption capacity at either lower or higher pH levels is due to the fact that, in the first stage of adsorption, the dye diffuses from the solution to the surface of the material. Too low a pH can seriously affect the solubility of the dye in water, while too high a pH can also hinder the binding of the adsorbent to the dye [30,31]. For CR, the optimal pH value for the Congo red adsorption performance of the LCA is 6. When pH is between 8 and 10, the adsorption performance of LCA for Congo red is improved. This is because, with the improvement in alkalinity, -COOH in hydrogel is deprotonated and tertiary amine is electrostatically adsorbed with CR, which increases the adsorption efficiency [43]. Under alkaline conditions, the solution contains a high concentration of hydroxyl and gives the surface of the adsorbent a negative charge, resulting in the competitive adsorption of anionic CR dye molecules and hydroxyl on the active site of the adsorbent, which leads to a decrease in the adsorption amount of CR.

In summary, when the amount of adsorbent is certain, the acidic solution at pH = 4 is more favorable for the adsorption of MG, and the weakly acidic solution at pH = 6 is more favorable for the adsorption of CR.

### 4.2. Effect of Adsorption Time on Adsorption Performance and Kinetic Analysis of Adsorption

**Figure 8:**
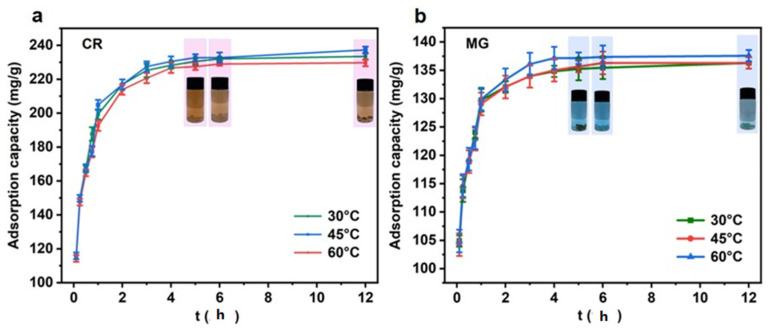
Effect of adsorption time at different temperatures on the adsorption of CR (**a**) and MG (**b**) by LCA.

The changes in the adsorption of CR and MG by the adsorbent at different times and at three temperature conditions, including 30 °C, 45 °C, and 60 °C, were investigated. As shown in Figure 8, the adsorption amounts of CR and MG gradually increase with the increase in time. When the adsorption time is 0–2 h, the adsorption amount increases rapidly. At this stage, there are a significant number of active sites on the adsorbent surface, and the dye molecules quickly migrate to these sites in the solution, which contributes to the rapid absorption of the dye. When the adsorption time is 0–2 h, the adsorption amount increases rapidly [12]. At this stage, there are a significant number of active sites on the adsorbent surfaces, and the dye molecules quickly migrate to these sites in the solution, which contributes to the rapid absorption of the dye. When the adsorption time reaches 2–4 h, the increased rate of adsorption gradually decreases, and most of the surface-active sites of the adsorbent at this stage are occupied by dye molecules. When the time reaches 4 h, the dye adsorption rate gradually levels off [44]. At this stage, dye molecules take more time to diffuse from the surface of the adsorbent to the internal pores, and the adsorption equilibrium state is not reached until 6 h. In addition, it can also be seen from the figure that for CR, within the whole reaction time, the adsorption capacity at 45 °C is basically greater than that at 60 °C and 30 °C. For MG, the curve at 60 °C is significantly higher than that at 45 °C and 30 °C, indicating that the adsorption capacity reaches its maximum at 60 °C. Therefore, the adsorption temperature of 45 °C is more conducive to the adsorption of CR, while the temperature of 60 °C is more conducive to the removal of MG.

The adsorption kinetic data of MG and CR were studied by using the quasi-first-order, quasi-second-order kinetic model, and intra-particle diffusion model, and the experimental data were processed by the linear fitting method.

The quasi-level kinetic model is as follows:

(3) $$\ln\left(q_{e}-q_{t}\right)=\ln q_{e}-k_{1}t$$

where:

$q_{e}$—equilibrium adsorption capacity, mg/g;
$k_{1}$—quasi-primary model adsorption rate constants, h^−1^;
$q_{t}$—the amount of adsorption in time *t*, mg/g.

The quasi-secondary dynamical model is as follows:

(4) $$\frac{t}{q_{t}}=\frac{1}{k_{2}q_{e}^{2}}+\frac{t}{q_{e}}$$

where:

$q_{e}$—equilibrium adsorption capacity, mg/g;
$k_{2}$—quasi-second-order model adsorption rate constant, g (mg·h)^−1^;
$q_{t}$—the amount of adsorbent per unit mass at any adsorption time, mg/g.

The particle diffusion equation is as follows:

(5) $$q_{t}=k_{d}t^{\frac{1}{2}}+C$$

where:

$q_{e}$—equilibrium adsorption volume, mg/g;
$k_{d}$—Weber–Morris adsorption rate constant, mg (g·h^1/2^)^−1^;
$q_{t}$—adsorption volume per unit mass of adsorbent at any adsorption time *t*, mg/g.

**Figure 9:**
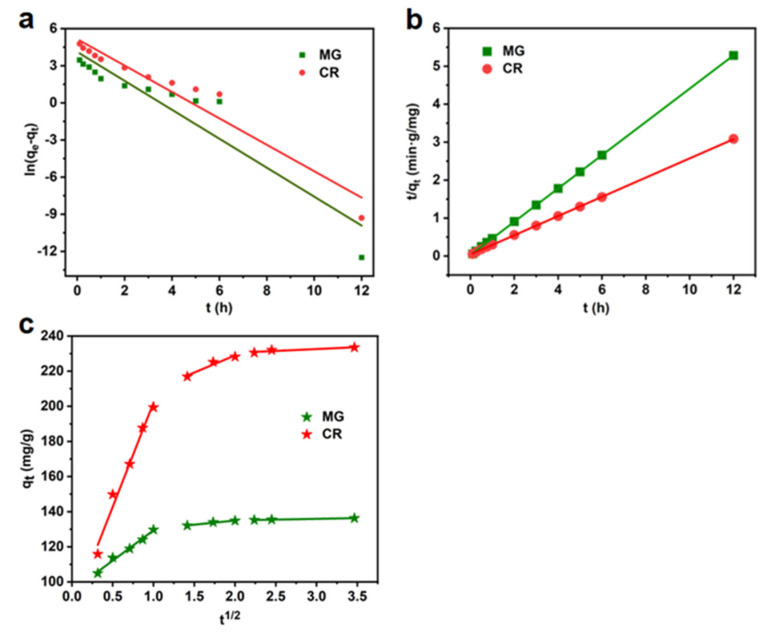
Quasi-primary kinetic model (**a**), quasi-secondary kinetic model (**b**), and intragranular diffusion model (**c**) for MG and CR.

The adsorption kinetic parameters for the three models are listed in Table 2 and Table 3. From Table 2, it can be seen that the adsorption dynamics of data fit better to the quasi-secondary kinetic model than to the quasi-first-order kinetic model for both CR and MG, with a higher R^2^. As shown in Figure 9, quasi-secondary adsorption kinetics proposes a bimolecular layer adsorption mechanism in which the surface functional groups of both the adsorbent and the adsorbate contribute to the rate of the chemisorption process [45,46], suggesting that the adsorption of CR and MG by the LCA is a bimolecular layer adsorption mechanism and that this mechanism is chemisorption. As can be seen from Table 4, K_i1_ > K_i2_ > K_i3_, indicating that the rapid adsorption of the two dyes in the initial stage of adsorption is mainly controlled by diffusion in the boundary layer, while the slow adsorption in the later stage is mainly controlled by intraparticle diffusion, and, the larger the K, the greater the influence of the boundary layer on the adsorption, i.e., the greater the influence of membrane diffusion on the adsorption process.

In summary, the removal of anions by the LCA is superior to that of cationic dyes. CR and MG and the LCA all contain conjugated structures and aromatic ring structures, which can form π-π interactions with each other via a benzene ring, thus adsorbing organic materials. Furthermore, the addition of lignin sulfonate increases the sulfonic acid group and hydroxyl group, adding more adsorption sites, and CR dye has a negatively charged sulfonic acid group, which can produce electrostatic attraction with the material. In summary, the adsorption of two dye ions by the LCA is mainly due to electrostatic interaction, hydrogen bonding, and π-π conjugation.

### 4.3. Adsorption Isotherm

Adsorption isotherms can explain the contact behavior of an adsorbate and adsorbent under equilibrium conditions, and the Langmuir and Freundlich models have been widely used to demonstrate adsorption equilibrium [47]. The Langmuir model assumes that adsorption occurs on the surface of a monolayer homogeneous adsorbent, and the Freundlich model is an empirical formulation that describes the multimolecular adsorption behavior of the adsorbent surface. The Langmuir and Freundlich isothermal sorption models are described as follows:

(6) $$\frac{C_{e}}{q_{e}}=\frac{C_{e}}{q_{e}}+\frac{1}{K_{L}q_{m}}$$

(7) $$\ln q_{e}=\ln K_{F}+\frac{\ln C_{e}}{n}$$

where:

$q_{e},C_{e}$—adsorption capacity at adsorption equilibrium, mg/g; the concentration of dye remaining at adsorption equilibrium, mg/L;
$q_{m}$—maximum adsorption capacity, mg/g;
$K_{L}$—affinity-related constants of binding sites in the Langmuir isothermal adsorption model, L/mg;
$K_{F},n$—Freundlich isothermal adsorption model adsorption equilibrium constants, $mg^{1-1/n}\cdot L^{-1/n}$; adsorption strength constant.

**Figure 10:**
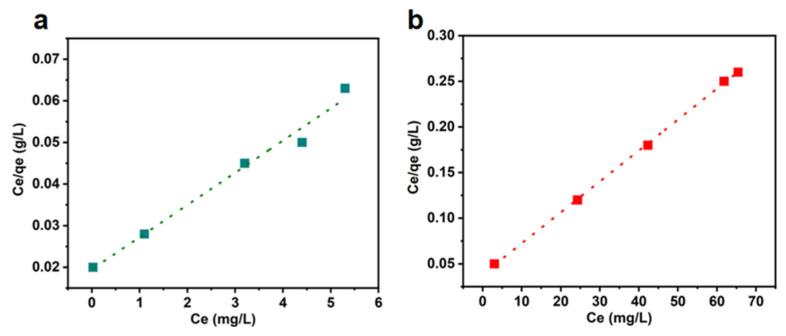
Linear fit of Langmuir model for MG (**a**) and CR (**b**).

**Figure 11:**
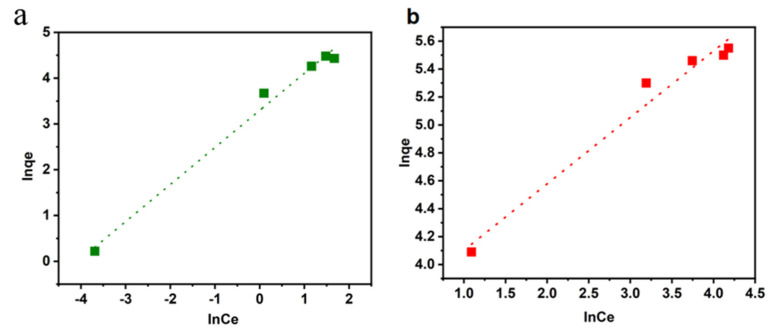
Linear fit of Freundlich model for MG (**a**) and CR (**b**).

The linear fitting of the Langmuir and Freundlich models is shown in Figure 10 and Figure 11. The fitting parameters are summarized in Table 4. The higher correlation coefficient (R^2^) indicates that the adsorption process of CR with the LCA was better fitted to the Langmuir isothermal adsorption model, while the adsorption process of MG was better fitted to the Freundlich isothermal adsorption model. Therefore, the adsorption process of CR is more monolayered, while that of MG is dominated by multi-molecular layer adsorption. The maximum adsorption capacity of LCA for CR and MG was 294.0 mg/g and 129.8 mg/g, respectively.

The solution temperature has an effect on the adsorption process and the adsorption efficiency of the LCA. Thermodynamic parameters such as Gibbs free energy (∆G), enthalpy change (∆H), and entropy change (∆S) are now used to explain the adsorption process:

(8) $$K_{d}=\frac{mq_{e}}{C_{e}V}$$

(9) $$\ln K_{d}=\frac{{∆S}^{0}}{R}-\frac{{∆H}^{0}}{RT}$$

(10) $${∆G}^{0}=∆H-T∆S$$

where:

R—standard molar constant, 8.314 × 10^−3^ J/(mol·K);
${∆G}^{0}$—Gibbs free energy, kJ/mol;
${∆S}^{0}$, ${∆H}^{0}$—standard entropy and enthalpy changes, kJ/mol;
$K_{d}$—allocation factor;
m—adsorbent quality, g;
V—volume of dye solution, L.

Table 5 shows the thermodynamic parameters for the adsorption of two dyes by the LCA. As can be seen from Table 5, in the adsorption of CR and MG, ${∆G}^{0}$ < 0 indicates that the adsorption process is a positive spontaneous process, and ${∆G}^{0}$ decreases with increasing temperature, indicating that higher adsorption equilibrium capacities are easily obtained at higher temperatures due to the conversion of heat into kinetic energy, which increases their adsorption performance [47]. ∆H > 0 indicates that this is a heat absorption ∆S process, and both are positive, indicating that the disorder is increasing at the solid–liquid interface between the adsorbent and the two dye solutions during the adsorption process.

## 5. Conclusions

Lignin/cellulose hydrogel materials were prepared from sodium lignosulfonate and cellulose, and 0.1% acetic acid solution was used as the solvent. The prepared hydrogel has a regular, non-collapsing three-dimensional network structure. The existence of pores greatly improved its water absorption and water retention. It has an excellent ability to adsorb large molecules, and its adsorption performance was investigated for two dye ions, CR and MG. The effects of solution pH, adsorption time, and temperature on the adsorption performance were also examined. The results showed that the best adsorption conditions for Congo red were an adsorption time of 4 h, a pH of 6, and an adsorption temperature of 45 °C. The adsorption process was in accordance with the Langmuir isothermal model and the quasi-secondary kinetic model and belonged to monomolecular layer adsorption. The maximum adsorption amount was 294.0 mg/g. The adsorption process was also in accordance with the Freundlich isothermal model and the quasi-secondary kinetic model and belonged to the chemisorption-dominated multilayer adsorption, with the maximum adsorption amount of 129.8 mg/g. According to the experimental results, we speculate that the hydrogel has great potential in the field of adsorbents, especially in the treatment of dye wastewater, and the hydrogel shows good adsorption performance.

## Figures and Tables

**Table 2:** Parameters related to quasi-primary kinetic model and quasi-secondary kinetic model for adsorption of CR and MG.

Samples	Quasi-Primary Adsorption Kinetic Model			Quasi-Secondary Adsorption Kinetic Model		
	$Q_{1}$ (mg·g^−1^)	$k_{1}$ (min^−1^)	R^2^	$Q_{2}$ (mg·g^−1^)	$k_{2}$ (g·mg^−1^·min^−1^)	R^2^
CR	172.43	0.0195	0.8656	237.15	4.27 × 10^−4^	0.9998
MG	136.30	1.0625	0.9298	136.92	1.98 × 10^−5^	0.9998

**Table 3:** Parameters related to the intra-particle diffusion model for adsorption of CR and MG.

Samples	$K_{i1}$ (mg·g^−1^·min^−0.5^)	$R_{1}^{2}$	$K_{i2}$ (mg·g^−1^·min^−0.5^)	$R_{2}^{2}$	$K_{i3}$ (mg·g^−1^·min^−0.5^)	$R_{3}^{2}$
CR	15.30	0.9738	0.61	0.9433	0.110	0.9985
MG	4.45	0.9842	2.52	0.9097	0.277	0.7518

**Table 4:** Parameters of Langmuir model (a) and Freundlich model (b) of Congo red and malachite green dyes.

Samples	Langmuir Adsorption Isotherm Model			Freundlich Adsorption Isotherm Model		
	$K_{L}$ (L/mg)	$Q_{m}$ (mg·g^−1^)	R^2^	$K_{F}$ $\left(mg^{1-1/n}\cdot L^{-1/n}\right)$	n	R^2^
CR	0.087	294.0	0.9957	37.33	2.13	0.9633
MG	0.39	129.8	0.9918	24.53	1.25	0.9944

**Table 5:** Thermodynamic parameters of adsorption of Congo red and malachite green by LCA.

Samples	$\Delta G^{0}/(KJ\cdot {mol}^{-1})$			$\Delta H^{0}/(KJ\cdot {mol}^{-1})$	$\Delta S^{0}(KJ\cdot {mol}^{-1})$
	30 °C	45 °C	60 °C		
CR	−15.81	−16.60	−17.38	1.08	16.53
MG	−8.49	−8.91	−9.33	0.896	22.53

## Data Availability

Data sharing is not applicable to this article as no datasets were generated or analyzed during the current study.

## Supplementary Materials

The following supporting information can be downloaded at: https://www.mdpi.com/article/10.3390/ma16124260/s1.
