# Supplementary material for: Preparation and Adsorption Properties of Lignin/Cellulose Hydrogel
Source: Materials (Basel). 2023 Jun 8;16(12):4260. doi: 10.3390/ma16124260 (PMC10303385; doi:10.3390/ma16124260)
Supplement: Supplementary file 1 [file materials-16-04260-s001.zip › materials-2376599-supplementary.pdf]

Supplementary Data for the article

Preparation and Adsorption Properties of Lignin/Cellulose Hydrogel

1. Characterization

1.1 Scanning electron microscope testing

The microscopic features of lignin hydrogels and lignin/cellulose hydrogels were observed using a quanta 20 field emission scanning electron microscope from Hitachi, Japan, and the powder samples were directly adhere to the conductive adhesive on the sample table before SEM testing, followed by gold spraying to eliminate static electricity.

1.2 FTIR spectrum

Samples were measured by KBr compression method on a German VERTEX 80V infrared spectrometer in the wavelength range of 4000-400 cm^{-1}.

1.3 Thermogravimetric

Adopt TGA209 F1 thermogravimetric analyzer from NETZSCH Germany to analyze the thermal stability performance of samples, protected by high purity nitrogen, experimental temperature 30-700℃, heating speed 10℃/min.

1.4 Analysis of nitrogen adsorption-desorption curves

The specific surface, pore volume and pore size distribution of lignin hydrogel and lignin/cellulose hydrogel materials were analyzed using a QUADRASORB-EVO gas adsorption instrument from USA. The thermostatic adsorption and desorption curves were analyzed to calculate the pore structure parameters such as specific surface area, pore volume and pore size distribution. The samples were prepared by pretreatment at 150°C for 8 h and then tested on a series of fully automated physical chemisorption instruments. The specific surface area was calculated by the multi-point method (brunauer-emmett-teller (BET)), and the pore volume and pore size distribution were calculated by the Barrett-Joyner-Halenda (BJH) method.

1.5 X-ray diffraction spectroscopy

The crystal structure analysis of the polymers was carried out on a multifunctional horizontal X-ray diffractometer of the Japanese Nikkei Ultima IV combination type. Test conditions: X-ray tube with CuKα target (λ= 0.15406 nm), graphite monochromator to eliminate CuKα radiation, tube voltage 40 kV, tube current 200 mA, scanning range 2θ =5~60°, scanning step 0.02°, scanning rate 15°/min, recording "diffraction intensity-2θ" curve.

1.6 X-ray photoelectron spectroscopy (XPS)

An AXIS Ultra DLD X-ray photoelectron spectrometer (XPS) from Shimadzu, Janpa was used to determine the surface chemical composition of the samples, including details of the surface elemental composition and peaks of C (C 1s), and S (S 2p). The sample concentration is greater than 0.1%, the detection depth is less than 10 nm, at least two samples are prepared for each material, and at least three different positions are measured for each sample. Including the relative content of elements and the type and relative content of chemical functional groups.

2. Adsorption of dyes

Maximum absorption wavelength determination: Congo red and malachite green were accurately weighed for the corresponding organic pollutants, accurately configured with 100 mg/L solution in volumetric flasks, and formulated into different concentration gradients. The absorption or reflectance in the visible range directly affects the perceived color of the chemicals involved, and the full spectral scan of the two organic pollutant solutions can be performed separately by UV spectrophotometer (UV2200, Shanghai Sunyu Hengping Scientific Instruments Co., Ltd.) to determine the maximum absorption wavelength of the dyes.

Standard curve determination: Congo red (20 mg/L, 40 mg/L, 60 mg/L, 80 mg/L, 100 mg/L)and malachite green (5 mg/L, 10 mg/L, 15 mg/L, 20 mg/L, 25 mg/L)were prepared in different concentration gradients, and the absorbance at the maximum absorption wavelength of the corresponding organic pollutant solutions were measured at different concentrations. The linear relationship between the absorbance of organic pollutants and the concentration of organic pollutants was fitted, and the merit of the fitted relationship was judged by the correlation coefficient R^2(controlled above 0.99). As a result, the standard curves of the organic pollutant solutions were obtained, and the basic information of the two organic dyes and the relevant parameters of the fitted standard curve equations are shown in Table S1.

Table S1. Basic parameters and fitting standard curve equation of three organic dyes

Name	Congo red	Malachite green
Molecular formula	$C_{32}H_{22}N_6Na_2O_6S_2$	$C_{23}H_{25}N_2Cl$
Molecular weight	696.68	364.5
Structural type	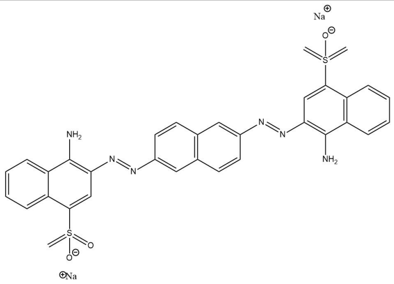	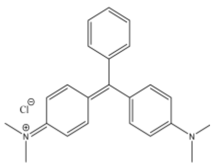
Type	Anionic type	Cationic type
Maximum absorption wave/nm	499	620
Standard	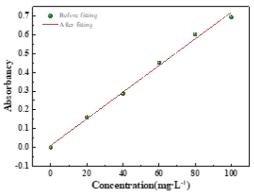	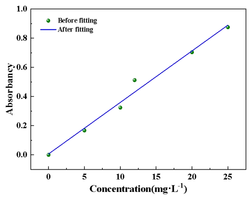

Standard curve equation	y=0.00709x+0.0111	y=0.0353x+0.0643
Correlation coefficient R^2	0.995	0.992
